# Differential effects of socio-demographic factors on maternal haemoglobin concentration in three sub-Saharan African Countries

**DOI:** 10.1038/s41598-020-78617-3

**Published:** 2020-12-07

**Authors:** Dickson A. Amugsi, Zacharie T. Dimbuene, Catherine Kyobutungi

**Affiliations:** 1 Maternal and Child Wellbeing Unit, African Population and Health Research Center, APHRC Campus, Box 10787-00100, Nairobi, Kenya; 2 Department of Population Sciences and Development, University of Kinshasa, Kinshasa, Democratic Republic of the Congo; 3 Social Analysis and Modeling Division, Statistics Canada, Ottawa, K1A 0T6 Canada

**Keywords:** Nutrition, Public health, Risk factors, Epidemiology

## Abstract

Low Haemoglobin concentration (Hb) among women of reproductive age is a severe public health problem in sub-Saharan Africa. This study investigated the effects of putative socio-demographic factors on maternal Hb at different points of the conditional distribution of Hb concentration. We utilised quantile regression to analyse the Demographic and Health Surveys data from Ghana, Democratic Republic of the Congo (DRC) and Mozambique. In Ghana, maternal schooling had a positive effect on Hb of mothers in the 5th and 10th quantiles. A one-year increase in education was associated with an increase in Hb across all quantiles in Mozambique. Conversely, a year increase in schooling was associated with a decrease in Hb of mothers in the three upper quantiles in DRC. A unit change in body mass index had a positive effect on Hb of mothers in the 5th, 10th, 50th and 90th, and 5th to 50th quantiles in Ghana and Mozambique, respectively. We observed differential effects of breastfeeding on maternal Hb across all quantiles in the three countries. The effects of socio-demographic factors on maternal Hb vary at the various points of its distribution. Interventions to address maternal anaemia should take these variations into account to identify the most vulnerable groups.

## Introduction

Maternal anaemia or low haemoglobin (Hb) concentration, a condition in which the Hb is lower than normal is a worldwide public health problem^1^. It is caused by deficiencies in iron, folate, copper, and other vital vitamins^2,3^. Also, infectious disease morbidity, parasitic infections and blood-related genetic disorders, among others, could cause low Hb concentration^3,4^. While the causes of low Hb concentration are multifaceted, the evidence shows that an estimated 50% of low Hb concentration cases reported worldwide are due to iron deficiency^5–7^. The available data suggest that anaemia affects about 500 million women of reproductive age, globally^8,9^**.** The World Health Organisation (WHO) estimates indicate that the global anaemia prevalence > 30%^2,6^. Consequently, the WHO included a target of reducing anaemia among women of reproductive age by 50% by 2025 in its Global Nutrition Targets (GNT)^5^. Similarly, anaemia was recently added as an indicator to track the progress of sub-goal 2.2 of the Sustainable Development Goals (SDGs) to end all forms of malnutrition by 2030^10^. It is significant to note that the problem of maternal low Hb concentration is particularly severe in sub-Saharan Africa (SSA) where poverty is highly prevalent and nutritious food is not easily accessible, coupled with a high incidence of infectious diseases^11–13^.

The consequences of low Hb concentration on the health of women include but are not limited to increased risks of low birth weight, preterm birth, perinatal mortality, and neonatal mortality^14^. Low Hb concentration also places women at an elevated risk of death during childbirth and postpartum^15^. Additionally, the literature suggests that low Hb concentration can result in cardiac decompensation (i.e. the failure of the heart to maintain adequate blood circulation). It also elevates the risk of Postpartum haemorrhage (PPH) and decreases the ability to tolerate blood loss, which can lead to circulatory shock and death^16,17^. The consequences of low Hb concentration mentioned above calls for an investigation that would examine the effects of putative factors across the conditional distribution of the Hb concentration. Such an inquiry will provide entry points for interventions to address maternal anaemia in developing countries. This study intends to achieve this goal by using an analytical strategy that focuses on the effects of socio-demographic factors at different stages of the conditional distribution of maternal Hb concentration.

The existing literature has identified several factors that have both negative and positive effects on maternal Hb concentration. Some of these factors include maternal age, education, parity, marital status, household size, socioeconomic status, place of residence, body mass index (BMI) and breastfeeding^18–25^. A study in Dhaka showed a strong relationship between maternal age, education level, income level, and maternal Hb concentration^18^. Moreover, higher BMI, primiparity, and living in better-off households were associated with higher levels of Hb^21,22^**.** On the contrary, low family income and large family size are negatively related to maternal Hb concentration^24^**.**Further, low maternal BMI, high parity, living in poorest wealth quintile and breastfeeding were negatively associated with maternal Hb concentration^25^. Other studies have shown that being separated or widowed, using an intrauterine device and being poor in rural and urban places of residence increased the odds of low Hb concentration among women^22,23^.

Indeed, from the studies reviewed above, it appears the literature on maternal anaemia abound. Nonetheless, there are shortfalls with the analytical strategies employed in these studies. For instance, almost all the referenced literature used either Ordinary Least Squares (OLS) or logistic regression to estimate the effects of socio-demographics on Hb concentration. This type of analysis may mask the different effects the socio-demographic factors may have on Hb concentration at the various points of its distribution. These possible differential effects suggest the need to undertake an analysis that has the potential to present a comprehensive picture of the effects of the putative factors on maternal Hb concentration. The quantile regression analytical strategy utilised in this paper can determine the effects of the socio-demographic factors at different points of the distribution of maternal Hb concentration. Therefore, the objective of this study was to examine the effects of socio-demographic factors on maternal Hb concentration using quantile regression. This type of analysis is currently missing in the anaemia research arena. It is worth noting that the word “maternal” used in this manuscript refers to non-pregnant mothers of children under five years. Therefore, the two terms may be used interchangeably.

## Methods

### Data sources and study design

This study involved a secondary analysis of the demographic and health survey (DHS) data^26^ from Ghana (2014), Mozambique (2011), and the Democratic Republic of Congo (DRC) (2013–2014). These are nationally representative data collected every five years in low and middle-income countries (LMICs). We based the selection of the three countries on our previous analysis, as well as the availability of Hb concentration data^27^. The earlier work involved an investigation of the correlates of the double burden of malnutrition (DBM) among women of reproductive age in five sub-Saharan African countries (Ghana, Kenya, Nigeria, Mozambique and DRC)^27^. Therefore, the present analysis was intended to build on this work, using the same countries. However, due to lack of Hb concentration data, we excluded two of the countries (Kenya and Nigeria).

In designing the surveys, the DHS ensured that the surveys are identical across all participating countries to facilitate comparison between and among nations. The DHS utilised a two-stage sample design in the selection of participating households in their surveys. The detail description of the DHS design processes is published elsewhere^28–32^. The questionnaires used in collecting the data as well as the training and interview protocols are the same across participating countries. Also, the DHS used standardised equipment in collecting anthropometric and biomarker data in all participating countries.

### Study participants

The study participants were mothers aged 15–49 years with at least one child under five years, and who had complete Hb concentration data. Information on study participants was obtained through face-to-face interviews with each participant. The DHS collected blood samples for anaemia testing from mothers who voluntarily consented to be tested^30^. Blood samples were drawn from a drop of blood taken from a finger prick and collected in a microcuvette. Hb concentration analysis was undertaken on-site using a battery-operated portable HemoCue analyser. Non-pregnant mothers with a Hb concentration of less than 7.0 g/dl were referred to a nearby health facility for immediate treatment^30^. The total samples of non-pregnant women per each country used in the present analysis were Ghana (n = 2975), DRC (n = 9438) and Mozambique (n = 10,961).

### Ethical statement

The DHS study was undertaken based on high ethical standards^33^. Data collectors were trained to recognise and respect the rights of study participants. They also informed participants of their rights to decide whether to participate in the study or not. The risks and benefits of the study, as well as steps taken to mitigate the potential risks, were adequately explained to study participants. The protocols of the study in each country, including biomarker collection, were approved by the recognised ethics review committees of each country, and the Institutional Review Board of ICF International, USA. Written informed consent was obtained from each study participant before they were allowed to participate in the study. However, study participants younger than 18 years, were made to give informed assent. At the same time, informed consent was obtained from their parent(s)/legal guardians before they were allowed to participate in the study. The biomarker results were made available to study participants^30^. The DHS Program, USA, granted permission to the authors for the use of the data. Due to the anonymous nature of the data, the authors did not seek further ethical clearance.

### Measures

#### Outcome variable

We used maternal Hb concentration (g/dl) as the outcome variable for this analysis. As described in the preceding sections, DHS collected blood samples from eligible women to test for anaemia using various strategies. The Hb concentration is captured first in the DHS data as a continuous variable and then categorised into three levels of anaemia: mild, moderate and severe. In this analysis, we used the Hb concentration as a continuous variable.

#### Predictor variables

We grouped the predictor variables into three main categories**:** *maternal* (education, age, BMI, employment status, parity, breastfeeding status, marital status and ANC attendance); *household* (wealth index, sex of household head, household size, number of children under five years, decision making on large household purchases and husband/partner education) and *community* (place of residence). The DHS created the household wealth index using assets ownership and housing characteristics: type of roofing, and flooring material, source of drinking water, sanitation facilities, ownership of television, bicycle, and motorcycle, automobile among others. The details of the computation processes are published elsewhere^30^**.** For the anthropometric data, adjustable measuring boards and electronic weighing scales were used to measure the participants’ height and weight, respectively. The maternal BMI (kg/m^2^) was then obtained by dividing weight in kilogrammes by height in meters squared and treated in the analysis as a continuous variable. The ANC attendance was categorised into ANC = 0–3 visits (reference) and ANC ≥ 4 visits.

### Data analysis

#### Outline of the quantile regression model

Koenker and Bassett^34^ introduced the quantile regression (QR) as a *location model* to extend Ordinary Least Squares (OLS). It is the case because OLS summarises the distribution at the grand mean. However, the QR assesses more general class of linear models in which, the conditional quantiles have a linear form to account for the overall distribution of the response variable fully. To formalise the QR, we consider a real-valued random variable *Y* characterised by the following distribution function^34,35^;

1 $${\text{F}}\left( y \right) \, = {\text{ Pr}}\left( {{\text{Y }} \le {\text{ y}}} \right)$$

Then for any *T* ϵ (0, 1), the *T*-th quantile of Y is defined as:

2 $${\text{Q }}\left( T \right) \, = {\text{ inf}}\left\{ {y:{\text{ F}}\left( y \right) \, \ge T} \right\}$$

The common quantiles *T *from Eq. (1) are *T* = 0.25, *T* = 0.50, and *T* = 0.75 for the first, the median and the third quartile. Therefore, unlike the OLS, which minimises the squared differences around the mean, QR minimises the weighted *absolute* difference between the observed value of y and the *T*-th quintile of Y. The preceding discussion demonstrates that OLS is nested within QR^34,35^.

### Analytical approach utilised

We used quantile regression (QR)^34^ to examine the effects of the putative socio-demographic factors on maternal/mothers Hb concentration. Using the QR, we were able to investigate the effects of the predictor variables at different points of the conditional distribution of the outcome variable (Hb concentration). This type of analysis cannot be done with OLS, because standard OLS regression techniques summarise the average relationship between a set of regressors and the outcome variable based on the conditional mean function E (y|x). Thus, it provides only a partial view of the relationship, as we might be interested in describing the relationship at different points in the conditional distribution of y. The QR, unlike OLS, provides a complete view of the effects of the predictor variable on the outcome variable. Thus, making it possible to identify the vulnerable groups that are in dire need of interventions. Further, QR is more robust in handling non-normal errors and outliers compared with OLS^34^. Finally, QR provides a richer characterisation of the data, thereby illuminating the effects of a covariate on the entire distribution of the outcome variable. In this analysis, we also included OLS estimates for comparison purposes, and estimated QR at the 5th, 10th, 25th, 50th, 75th and 90th quantiles^27,35^.

Furthermore, since we did not have a specific predictor variable of interest, all the socio-demographic variables were included simultaneously in the models. They include maternal education, age, BMI, employment status, parity, breastfeeding status, marital status, ANC attendance, household wealth index, sex of household head, household size, number of children under five years, decision making on large household purchases, husband/partner education and place of residence. The variables outlined above were selected based on the literature, followed by bivariate analysis. Significant variables in the bivariate analysis were included in the QR models. Because the DHS used complex survey design (CSD), we adjusted for design effects in all the analyses, using the *svyset* and *svy* procedures in STATA.

## Results

### Descriptive analysis of the characteristics of samples

The descriptive results (Table 1) showed that mean maternal Hb concentration was relatively the same across all the three countries: Ghana (11.95 ± 1.49), DRC (12.05 ± 1.65) and Mozambique (11.64 ± 1.73). Mothers in Ghana (5.27 ± 4.92) spent a little more years in education than those in DRC (4.88 ± 3.77) and Mozambique (3.46 ± 3.45). The mean age of the study participants ranged from 28 years in Mozambique to 31 years in Ghana. Moreover, mothers in Ghana tended to have higher mean BMI (24.34 ± 4.96) relative to those in DRC (21.79 ± 3.66) and Mozambique (22.53 ± 3.70). In DRC, 68% of mothers indicated they were breastfeeding at the time of the study, while the number of breastfeeding mothers in Ghana and Mozambique stood at 58%, respectively.

**Table 1:** Characteristics of the socio-demographic variables of the three countries.

Variables	Ghana (n = 2975)		DRC (n = 9438)		Mozambique (n = 10,961)	
	Mean/%	SD	Mean/%	SD	Mean/%	SD
**Maternal-level variables**						
Hb concentration (g/dl)	11.95	1.49	12.05	1.65	11.64	1.73
Women education (in years)	5.27	4.92	4.88	3.77	3.46	3.45
Age (in years)	30.65	6.96	29.13	6.94	28.38	7.18
Women's body mass index (BMI) (kg/m^2^)	24.34	4.96	21.79	3.66	22.53	3.70
Working (yes)	79.3		76.0		37.5	
Parity	3.62	2.17	4.42	2.56	3.8	2.28
Breastfeeding (yes)	57.9		67.8		57.6	
*Marital status*						
Never married	6.4		4.2		5.6	
Married or cohabiting	87.4		87.1		82.8	
Divorced/widowed/separated	6.2		8.7		11.6	
**Household-level variables**						
*Wealth index*						
Poorest	33.0		27.4		18.2	
Poor	21.1		22.9		18.9	
Middle	19.0		20.7		19.7	
Rich	15.1		17.1		22.1	
Richest	12.3		12.1		21.1	
Head of household (Female)	23.7		22.2		32.9	
Household size	5.80	2.82	6.74	2.86	6. 16	2.79
Number of children under five years	1.73	0.93	2.16	1.04	1.86	0.98
*Decision on large household purchases*						
Respondent alone	16.0		12.5		11.1	
Respondent and husband/partner	46.5		50.3		57.1	
Husband/partner alone	24.1		36.7		30.7	
Someone else/Other	13.4		0.59		1.1	
Husband education (in years)	7.04	5.44	8.53	4.11	4.28	3.95
**Community-level variables**						
Place of residence (% urban)	39.8		28.7		32.1	

SDs are reported only for continuous variables.

*SD* standard deviation, *Hb* haemoglobin.

### Quantile multivariable regression analysis of the effects of socio-demographic factors on maternal Hb concentration

In Tables 2, 3 and 4, we present the QR results of the effects of socio-demographic factors on maternal Hb concentration in Ghana, DRC and Mozambique. We also reported the OLS estimates for comparison with the QR results. The results from the OLS analysis showed that maternal education had strong positive effects on Hb concentration in Ghana and Mozambique. Thus, in both countries, a one-year increase in education was associated with positive changes in maternal Hb concentration. Similarly, there was a positive association between maternal BMI and Hb concentration in all the three countries, so was breastfeeding practices.

**Table 2:** Effects of socio-demographic factors on maternal Hb concentration in Ghana.

Variables	OLS	Q5	Q10	Q25	Q 50	Q75	Q90
**Maternal level variables**							
Women education (in years)	0.015* (0.008)	0.063*** (0.016)	0.044** (0.016)	0.024 (0.013)	0.004 (0.010)	− 0.004 (0.009)	− 0.007 (0.015)
Age (in years)	0.009 (0.006)	0.013 (0.011)	0.024 (0.013)	0.012 (0.011)	0.006 (0.008)	− 0.005 (0.008)	− 0.001 (0.009)
Women's body mass index (BMI) (kg/m^2^)	0.020** (0.006)	0.037** (0.012)	0.022* (0.010)	0.009 (0.009)	0.019* (0.008)	0.012 (0.008)	0.024* (0.010)
Mother working-No (reference)							
Mother working-Yes	0.121 (0.070)	0.008 (0.139)	0.003 (0.147)	0.129 (0.111)	0.142 (0.079)	0.095 (0.084)	0.159 (0.106)
Parity	− 0.002 (0.021)	− 0.009 (0.060)	− 0.014 (0.064)	0.019 (0.035)	0.009 (0.031)	0.036 (0.030)	0.036 (0.029)
Breastfeeding-No (reference)							
Breastfeeding-Yes	0.216*** (0.060)	0.435*** (0.132)	0.424*** (0.128)	0.442*** (0.106)	0.252** (0.077)	− 0.099 (0.085)	− 0.070 (0.101)
*Marital status*							
Never married (reference)							
Married or cohabiting	− 0.180 (0.328)	− 0.306 (0.650)	− 0.416 (0.766)	− 0.432 (0.523)	− 0.435 (0.504)	0.141 (0.491)	− 0.176 (0.573)
Divorced/widowed/separated	0.154 (0.159)	0.507 (0.406)	0.189 (0.330)	0.413 (0.214)	0.084 (0.176)	0.138 (0.193)	− 0.384 (0.266)
Antenatal visits = 0–3 (reference)							
Antenatal visits = 4 +	0.212* (0.093)	0.458 (0.247)	0.383 (0.214)	0.121 (0.167)	0.193 (0.121)	0.094 (0.131)	0.300* (0.138)
**Household-level variables**							
Wealth index-poorest (reference)							
Wealth index-Poor	− 0.199* (0.082)	− 0.243 (0.177)	− 0.411* (0.180)	− 0.169 (0.141)	− 0.188 (0.098)	− 0.136 (0.096)	− 0.071 (0.145)
Wealth index-Middle	− 0.128 (0.096)	0.012 (0.198)	0.109 (0.191)	− 0.076 (0.156)	− 0.303** (0.117)	− 0.079 (0.130)	0.100 (0.176)
Wealth index-Rich"	0.122 (0.119)	0.123 (0.266)	0.068 (0.257)	0.348 (0.195)	0.146 (0.128)	0.149 (0.135)	0.210 (0.228)
Wealth index-Richest	0.109 (0.141)	0.112 (0.302)	0.294 (0.300)	0.228 (0.245)	0.088 (0.149)	0.317 (0.176)	0.159 (0.229)
Head of household-Male (reference)							
Head of household-Female	0.067 (0.075)	0.016 (0.188)	0.149 (0.174)	0.102 (0.119)	0.005 (0.095)	− 0.004 (0.091)	0.035 (0.109)
Household size	0.011 (0.013)	0.001 (0.045)	0.031 (0.033)	0.019 (0.017)	0.004 (0.014)	0.000 (0.017)	− 0.003 (0.017)
Number of children under five	− 0.060 (0.038)	− 0.001 (0.108)	0.053 (0.094)	− 0.095 (0.058)	− 0.128** (0.045)	− 0.087 (0.053)	0.006 (0.058)
*Decision on large household purchases*							
Respondent alone (reference)							
Respondent and husband/partner	0.232** (0.080)	0.227 (0.237)	0.268 (0.190)	0.247* (0.124)	0.296** (0.106)	0.120 (0.096)	0.160 (0.116)
Husband/partner alone	0.269** (0.088)	0.480* (0.236)	0.294 (0.203)	0.379** (0.121)	0.357** (0.118)	0.098 (0.101)	− 0.033 (0.144)
Someone else/other	0.008 (0.317)	− 0.281 (0.617)	− 0.153 (0.763)	− 0.397 (0.501)	− 0.158 (0.500)	0.227 (0.470)	0.252 (0.550)
Husband education (in years)	− 0.002 (0.007)	− 0.033* (0.014)	− 0.025 (0.016)	− 0.001 (0.011)	0.013 (0.009)	0.001 (0.008)	− 0.006 (0.011)
Place of residence-rural (reference)							
Place of residence-Urban	− 0.097 (0.077)	− 0.128 (0.202)	− 0.037 (0.175)	− 0.041 (0.111)	− 0.209** (0.081)	− 0.181* (0.090)	− 0.096 (0.140)
Observations	2975	2975	2975	2975	2975	2975	2975

Standard errors in parentheses.

*OLS* ordinary least squares, *Q* quantile.

*p < 0.05; **p < 0.01; ***p < 0.001.

**Table 3:** Effects of socio-demographic factors on maternal Hb concentration in DRC.

Variables	OLS	Q5	Q10	Q25	Q 50	Q75	Q90
**Maternal level variables**							
Women education (in years)	− 0.007 (0.006)	0.016 (0.018)	− 0.007 (0.013)	0.006 (0.008)	− 0.015** (0.006)	− 0.020** (0.007)	− 0.023* (0.011)
Age (in years)	0.004 (0.004)	− 0.010 (0.012)	− 0.007 (0.010)	0.006 (0.005)	0.012* (0.005)	0.005 (0.005)	0.003 (0.006)
Women's body mass index (BMI) (kg/m^2^)	0.015** (0.005)	0.026*** (0.008)	0.021** (0.007)	0.014* (0.006)	0.007 (0.005)	0.009 (0.007)	0.001 (0.010)
Mother working-No (reference)							
Mother Working-Yes	− 0.033 (0.041)	0.060 (0.107)	0.046 (0.082)	− 0.010 (0.052)	− 0.037 (0.044)	0.030 (0.051)	− 0.038 (0.070)
Parity	0.016 (0.012)	0.052 (0.037)	0.041 (0.026)	0.017 (0.018)	0.003 (0.014)	0.014 (0.016)	0.023 (0.018)
Mother breastfeeding-No (reference)							
Mother breastfeeding-Yes	0.470*** (0.038)	0.745*** (0.117)	0.662*** (0.079)	0.686*** (0.057)	0.488*** (0.045)	0.355*** (0.057)	0.219*** (0.061)
*Marital status*							
Never married (reference)							
Married or cohabiting"	− 0.128 (0.092)	− 0.219 (0.244)	− 0.372* (0.169)	− 0.186 (0.127)	− 0.242** (0.092)	− 0.145 (0.103)	0.108 (0.132)
Divorced/widowed/separated	0.071 (0.103)	− 0.010 (0.307)	− 0.060 (0.191)	− 0.026 (0.141)	0.007 (0.106)	0.179 (0.126)	0.320* (0.149)
Antenatal visits = 0–3 (reference)							
Antenatal visits = 4 +	0.025 (0.039)	0.011 (0.098)	0.027 (0.078)	0.030 (0.053)	0.014 (0.043)	0.016 (0.045)	0.095 (0.063)
**Household-level variables**							
Wealth index-Poorest (reference)							
Wealth index-Poor	− 0.030 (0.048)	0.024 (0.158)	0.091 (0.116)	− 0.062 (0.074)	− 0.056 (0.048)	− 0.081 (0.061)	− 0.048 (0.085)
Wealth index-Middle	0.104* (0.050)	0.219 (0.137)	0.282* (0.118)	0.106 (0.070)	0.058 (0.054)	− 0.011 (0.068)	0.027 (0.087)
Wealth index-Rich	0.064 (0.058)	0.279 (0.148)	0.393** (0.137)	0.112 (0.082)	− 0.004 (0.061)	− 0.106 (0.073)	− 0.020 (0.090)
Wealth index-Richest	− 0.062 (0.079)	0.192 (0.241)	0.206 (0.168)	− 0.072 (0.114)	− 0.207* (0.095)	− 0.039 (0.096)	− 0.070 (0.125)
Head of household-Male (reference)							
Head of household-Female	0.019 (0.045)	0.278* (0.136)	0.249** (0.083)	0.211*** (0.057)	− 0.033 (0.046)	− 0.129* (0.057)	− 0.179** (0.068)
Household size	0.001 (0.008)	0.035 (0.020)	0.001 (0.016)	− 0.004 (0.012)	− 0.002 (0.011)	0.002 (0.010)	− 0.009 (0.011)
Number of children under five	− 0.015 (0.021)	− 0.107 (0.056)	− 0.051 (0.053)	0.021 (0.031)	− 0.037 (0.023)	− 0.042 (0.026)	0.020 (0.035)
*Decision on large household purchases*							
Respondent alone (reference)							
Respondent and husband/partner	− 0.119* (0.056)	− 0.287 (0.148)	− 0.159 (0.114)	0.042 (0.077)	− 0.123 (0.069)	− 0.149* (0.064)	− 0.069 (0.102)
Husband/partner alone	− 0.195*** (0.056)	− 0.235 (0.159)	− 0.243* (0.101)	− 0.103 (0.078)	− 0.250*** (0.067)	− 0.131 (0.067)	− 0.156 (0.104)
Someone else/other	0.044 (0.224)	0.449 (0.261)	0.222 (0.263)	− 0.448 (0.559)	0.365 (0.217)	− 0.018 (0.313)	− 0.065 (0.185)
Husband education (in years)	0.005 (0.005)	− 0.005 (0.015)	0.002 (0.012)	− 0.010 (0.007)	0.006 (0.005)	0.011 (0.006)	0.019* (0.009)
Place of residence-rural (reference)							
Place of residence-urban	− 0.004 (0.051)	− 0.124 (0.138)	− 0.132 (0.108)	0.027 (0.069)	0.131** (0.049)	0.018 (0.056)	− 0.140 (0.093)
Observations	9438	9438	9438	9438	9438	9438	9438

Standard errors in parentheses.

*OLS* ordinary least squares, *Q* quantile.

*p < 0.05; **p < 0.01; ***p < 0.001.

**Table 4:** Effects of socio-demographic factors on maternal Hb concentration in Mozambique.

Variables	OLS	Q5	Q10	Q25	Q 50	Q75	Q90
**Maternal level variables**							
Women education (in years)	0.035*** (0.007)	0.055** (0.021)	0.043** (0.015)	0.030** (0.011)	0.037*** (0.008)	0.046*** (0.009)	0.027* (0.011)
Age (in years)	0.001 (0.004)	0.002 (0.009)	0.001 (0.007)	0.001 (0.006)	0.005 (0.004)	0.000 (0.005)	0.001 (0.005)
Women's Body Mass Index (BMI) (kg/m^2^)	0.019*** (0.005)	0.031* (0.015)	0.033** (0.011)	0.029*** (0.007)	0.018** (0.006)	0.008 (0.007)	0.008 (0.008)
Mother working-No (reference)							
Mother working-Yes	− 0.049 (0.034)	0.004 (0.094)	− 0.038 (0.071)	− 0.092 (0.052)	0.001 (0.039)	− 0.013 (0.042)	− 0.080 (0.052)
Parity	0.025* (0.012)	0.069* (0.027)	0.061** (0.024)	0.024 (0.015)	0.003 (0.011)	0.023 (0.017)	0.004 (0.018)
Mother breastfeeding-No (reference)							
Mother breastfeeding-Yes	0.424*** (0.036)	0.732*** (0.100)	0.663*** (0.077)	0.520*** (0.055)	0.459*** (0.041)	0.310*** (0.051)	0.219*** (0.054)
*Marital status*							
Never married (reference)							
Married or Cohabiting	− 0.067 (0.087)	− 0.445* (0.210)	− 0.275 (0.160)	− 0.159 (0.136)	0.139 (0.106)	− 0.055 (0.121)	0.075 (0.134)
Divorced/widowed/separated	− 0.139 (0.092)	− 0.495* (0.220)	− 0.221 (0.205)	− 0.144 (0.149)	− 0.062 (0.113)	− 0.138 (0.121)	− 0.005 (0.141)
Antenatal visits = 0–3 (reference)							
Antenatal visits = 4 +	0.049 (0.036)	0.045 (0.086)	0.028 (0.067)	0.035 (0.048)	0.015 (0.038)	0.036 (0.041)	0.075 (0.051)
**Household-level variables**							
Wealth index-Poorest (reference)							
Wealth index-Poor	0.255*** (0.054)	− 0.114 (0.157)	0.198 (0.110)	0.174* (0.076)	0.285*** (0.063)	0.326*** (0.063)	0.429*** (0.074)
Wealth index-Middle	0.367*** (0.054)	0.246 (0.131)	0.397*** (0.109)	0.249*** (0.073)	0.335*** (0.055)	0.399*** (0.067)	0.552*** (0.075)
Wealth index-Rich	0.380*** (0.056)	0.242 (0.146)	0.406*** (0.118)	0.268*** (0.079)	0.337*** (0.059)	0.400*** (0.067)	0.594*** (0.085)
Wealth index-Richest	0.257*** (0.073)	− 0.122 (0.191)	0.036 (0.150)	0.147 (0.109)	0.198** (0.074)	0.374*** (0.098)	0.474*** (0.111)
Head of household-Male (reference)							
Head of household-Female	− 0.010 (0.039)	− 0.061 (0.100)	− 0.150 (0.083)	− 0.040 (0.057)	0.075 (0.045)	− 0.050 (0.047)	− 0.074 (0.067)
Household size	0.015 (0.008)	0.014 (0.016)	0.007 (0.016)	0.004 (0.012)	0.028*** (0.008)	0.007 (0.011)	0.011 (0.017)
Number of children under five	− 0.025 (0.022)	− 0.077 (0.054)	− 0.070 (0.051)	0.001 (0.035)	− 0.053* (0.024)	0.015 (0.034)	0.003 (0.034)
*Decision on large household purchases*							
Respondent alone (reference)							
Respondent and Husband/Partner	0.010 (0.056)	− 0.146 (0.143)	− 0.092 (0.106)	− 0.050 (0.084)	0.075 (0.064)	0.027 (0.062)	0.088 (0.093)
Husband/Partner alone	− 0.032 (0.059)	− 0.021 (0.149)	− 0.061 (0.112)	− 0.031 (0.088)	− 0.050 (0.065)	− 0.020 (0.069)	0.043 (0.099)
Someone else/Other	0.244 (0.168)	0.817 (0.605)	0.349 (0.230)	0.133 (0.267)	0.062 (0.227)	0.430* (0.172)	0.314 (0.260)
Husband education (in years)	0.011* (0.006)	− 0.002 (0.015)	0.019 (0.012)	0.015 (0.008)	0.013 (0.006)	0.002 (0.007)	0.011 (0.010)
Place of residence-Rural (reference)							
Place of residence-urban	− 0.059 (0.046)	− 0.278* (0.129)	− 0.203* (0.088)	− 0.184** (0.067)	0.019 (0.046)	− 0.021 (0.058)	0.139 (0.072)
Observations	10,961	10,961	10,961	10,961	10,961	10,961	10,961

Standard errors in parentheses.

*OLS* ordinary least squares, *Q* quantile.

*p < 0.05; **p < 0.01; ***p < 0.001.

However, in the QR analysis (Tables 2, 3 and 4), the results revealed vital differences in effects at different points in the conditional distribution of the Hb concentration. For example, in Ghana, the effect of maternal years of education occurred at the first two lowest quantiles (5th and 10th), with the largest effect at the 5th quantile. Similarly, in Mozambique, a one-year increase in maternal education was associated with increases in Hb concentration across all quantiles, with the largest effect on mothers in the lowest quantile (5th) and the smallest effect at the highest quantile (90th). Interestingly, in DRC, maternal years of education had an inverse relationship with Hb concentration of mothers in the three upper quantiles. Thus, a one-year increase in maternal years of education was associated with 0.015, 0.020 and 0.023 units decrease in Hb concentration of mothers in the 50th, 75th and 90th quantiles, respectively. In Ghana, BMI had a significant positive effect on Hb concentration of mothers in the 5th, 10th, 50th and 90th quantiles. The effect on the remaining two quantiles did not reach statistical significance. The positive effects of BMI on Hb concentration was among mothers in the three lowest quantiles (5th, 10th and 25th) in DRC, while in Mozambique, a unit increase in maternal BMI was associated with 0.031, 0.033, 0.029 and 0.018 units increase in maternal Hb concentration at the 5th, 10th, 25th and 50th quantiles, respectively. In each of the countries, the largest effect of BMI on Hb concentration occurred at the lower end of the Hb distribution.

In Ghana, breastfeeding was positively and significantly associated with Hb concentration of mothers in the first four quantiles (5th, 10th, 25th, and 50th), with the least effect occurring at the 50th quantile. However, in DRC and Mozambique, breastfeeding had a decreasing effect across all quantiles in the respective countries. The largest effect in each country was at the lower end of the conditional distribution of the Hb concentration, while the smallest effect was at the higher end of the distribution. In Ghana, women participation in decision making regarding large household purchases was associated with a better Hb concentration among mothers in the 25th and 50th quantiles, while the partner taking the decision alone was associated positively with the Hb concentration at 5th, 25th and 50th quantiles. On the contrary, there was an inverse effect of the partner alone, deciding on large household purchases on Hb concentration of mothers in the 25th and 50th quantiles in DRC. The effects of female household headship in DRC was mixed. It associated positively with Hb concentration of mothers in the first three quantiles (5th, 10th and 25th), and negatively with the two upper quantiles (75th and 90th). In Mozambique, the household wealth index had a significant and increasing (i.e. from 5 to 90th) effect on maternal Hb concentration across almost all the quantiles. The largest effect occurred at the highest end of the Hb distribution (90th quantile). In Ghana, being in the lower wealth index was associated with a low Hb concentration among mothers in the 5th and 10th quantiles.

**Figure 1:**
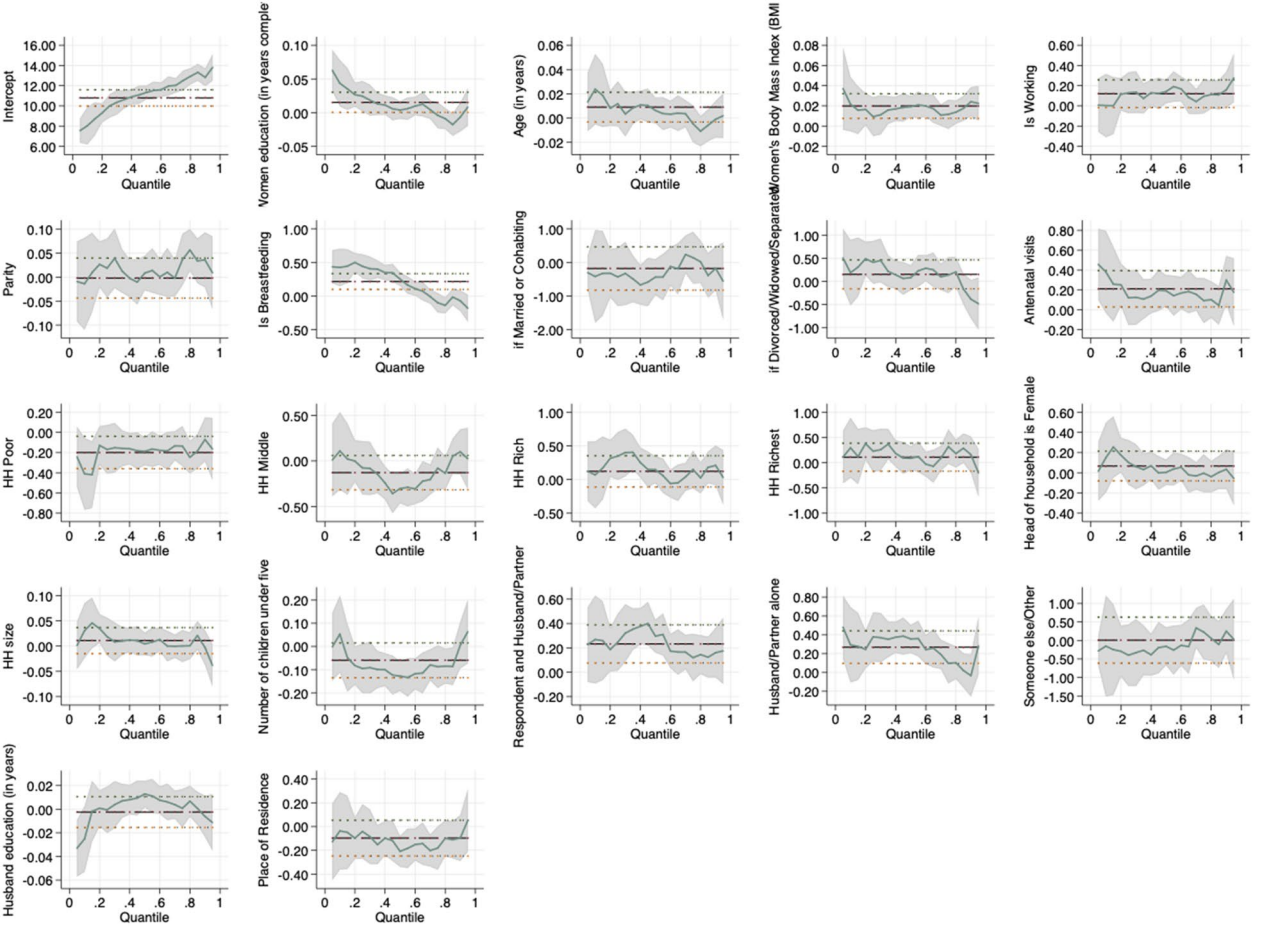
Pictorial presentation of the effects of socio-demographic factors on maternal Hb concentration in Ghana.

**Figure 2:**
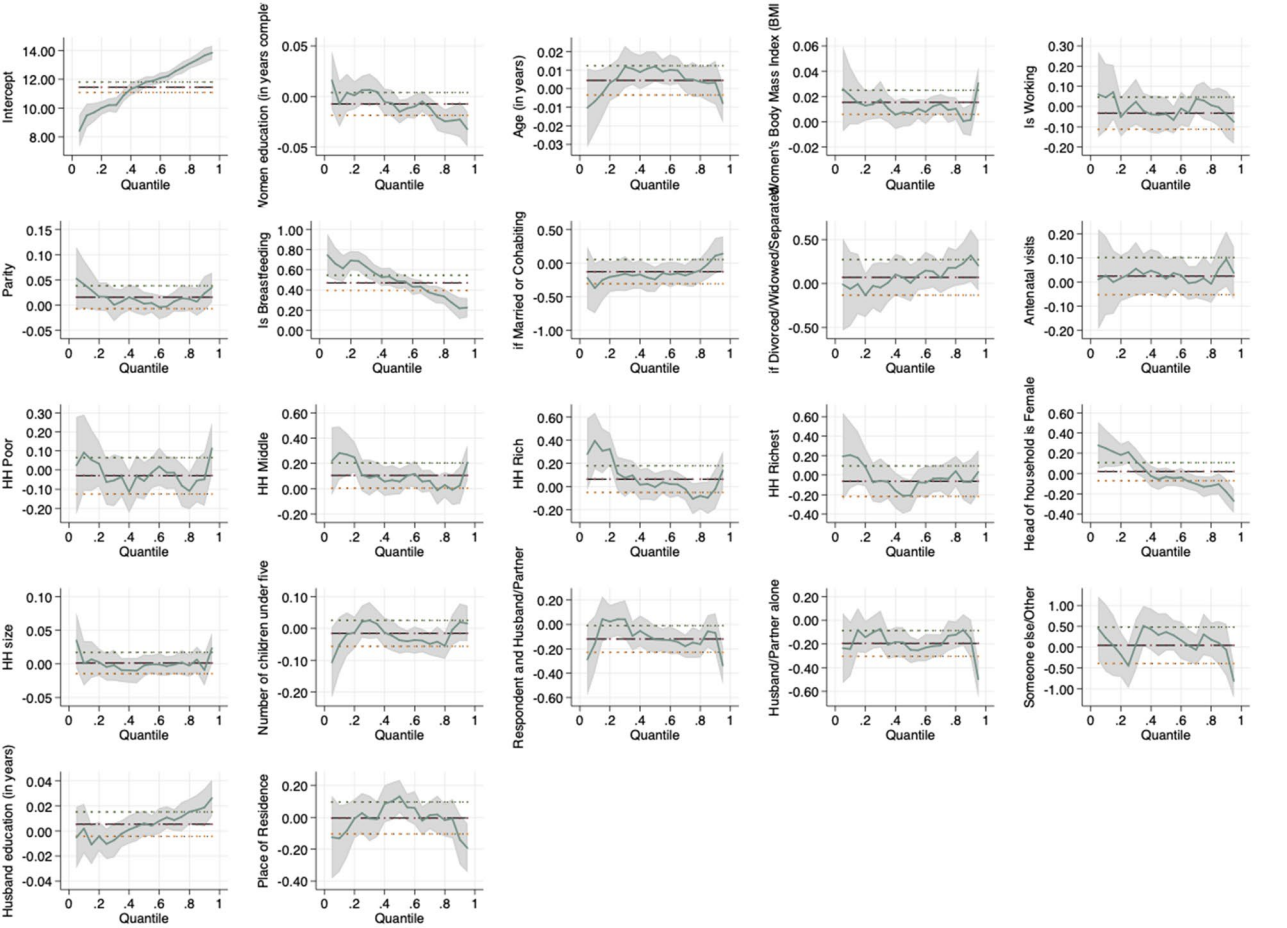
Pictorial presentation of the effects of socio-demographic factors on maternal Hb concentration in DRC.

**Figure 3:**
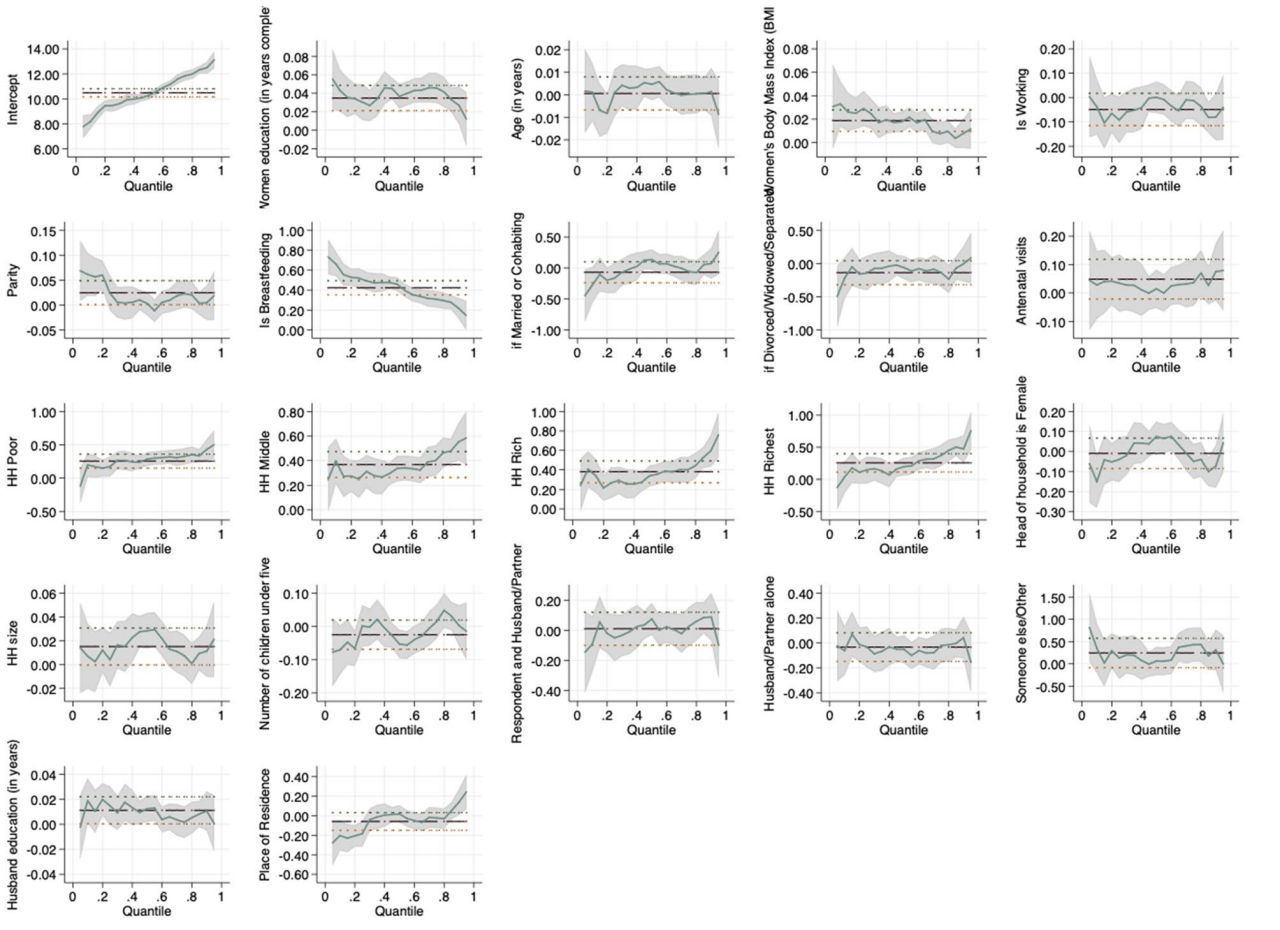
Pictorial presentation of the effects of socio-demographic factors on maternal Hb concentration in Mozambique.

Figures 1, 2 and 3 are visual presentations of the effects of the various putative socio-demographic factors on maternal Hb concentration in the three countries included in the analysis.

## Discussion

We investigated the effects of putative socio-demographic factors on maternal Hb concentration in Ghana, DRC and Mozambique, using quantile regression to understand the differential effects of these factors at different points of the conditional distribution of the Hb concentration. Our QR results showed that in Ghana, a one-year increase in maternal education had a significant positive effect on Hb concentration of mothers in the 5th and 10th quantiles. In contrast, the effects on the other four quantiles did not reach statistical significance. However, the OLS results suggest that a year increase in schooling had a positive effect on the Hb concentration of all mothers. The OLS results paint just a part of the picture and therefore, can be misleading. In Mozambique, we observed that the positive effect of years of schooling on maternal Hb was across all quantiles and in a decreasing manner. Implying that the largest effect of education occurred at the lowest quantiles, while the smallest effect was on Hb concentration of mothers in the highest quantile. Our findings in the two countries suggest disproportionate positive effects of maternal education accruing to mothers in the lower tail of the Hb distribution. Consequently, improving women education may be more impactful on the Hb concentration of mothers in the lower than the upper quantiles.

In contrast, we observed an inverse relationship between maternal years of schooling and the Hb concentration of mothers in the three upper quantiles in DRC. This finding implies that education appears to have non-beneficial effects on the Hb of mothers in the upper quantiles in DRC. This may be puzzling as the literature suggests that education consistently predict positive health outcomes in women^18,25,36,37^. For example, a study using multi-country data concluded that women with higher years of education were less likely to be anaemic relative to those with fewer years of schooling^36^. Further research is needed to elucidate the possible factors accounting for the negative effect of education on maternal Hb outcomes in DRC. Our study together with the literature, despite using different analytical strategies, strongly suggest that education has positive effects on maternal health outcomes.

Our analysis also showed that maternal BMI has a significant positive effect on Hb concentration in at least three quantiles in each country. The most significant effect of BMI was among mothers in the lower quantiles. Thus, suggesting that interventions targeted at improving women BMI qualitatively are likely to be more effective in increasing the Hb concentration of mothers in the lower tail of the Hb distribution. It is worthy to note that the effects of BMI were not across all quantiles. Hence, the OLS estimates, which suggested that maternal BMI positively associated with Hb concentration among all mothers, may be misleading. The QR findings are, therefore, critical for identifying the groups that need to be targeted in programme planning. The literature corroborated the results of our study. Several studies using either linear or logistic regression analytical strategies suggested that women with higher BMI tend to have higher levels of Hb concentration^22,23,36^.

Similarly, we observed a significant positive effect of breastfeeding on maternal Hb concentration in all the three countries. Mothers who were breastfeeding at the time of the survey tended to have better Hb concentration compared with non-breastfeeding mothers. The largest effects were observed among mothers in the lower quantiles, suggesting that interventions to promote breastfeeding among lactating mothers may have more impact on Hb concentration of mothers at the lower end of the Hb distribution. These findings may appear puzzling because it is generally believed that lactating mothers tend to lose some iron to their infants, which may have a bearing on their Hb concentration^38,39^. Nevertheless, other evidence suggests that the iron contained in breast milk to children is not significant enough to deplete the iron level of the mother unless the mother is already anaemic^40^. The literature further suggests that mothers who are anaemic postpartum can recover through a high intake of iron-rich diet and/or iron supplement, and may not suffer low Hb concentration during lactation^40–42^. The preceding discussion suggests that breastfeeding may not necessarily deplete maternal iron level, with the consequential adverse effect on Hb concentration. Some available evidence suggests a positive effect of breastfeeding on maternal Hb^38^. Nonetheless, other studies have observed inverse relationships between breastfeeding and Hb concentration levels^39,43^. These mixed findings notwithstanding, the results in the present study suggest that breastfeeding can indeed have positive effects on maternal Hb concentration levels. However, the mechanism through which this happens may be complicated.

Our findings in Mozambique suggest that household wealth index (HWI) had a positive and increasing effect on Hb concentration across all quantiles. The smallest effect was observed among mothers in the lower end of the Hb distribution, while the largest effect was on mothers at the upper end of the distribution. Thus, improving HWI may be more impactful on mothers at the upper quantiles relative to those at the lower quantiles. The positive association between HWI and women health outcomes have been substantially documented^38,44,45^. The evidence is that mothers who live in better-off households tend to have higher levels of Hb concentration^38^. However, in Ghana, mothers who live in poor households and are in the 5th and 10th quantiles tended to have lower Hb concentration. The finding in Ghana is consistent with the literature, which often identifies poverty as a risk factor of maternal health outcomes^25,46^.

We also observed that a unit change in maternal parity had a significant positive effect on the Hb concentration of mothers in the two lower quantiles (5th and 10th) in Mozambique. These findings are inconsistent with some previous studies which suggest that higher parity is associated with increased odds of anaemia in women of reproductive age^25,47–49^. For example, a study in Ethiopia observed that lactating mothers who had three or more births were at higher risk of anaemia relative mothers who had one birth^47^. However, it is significant to point out that the present study did not treat parity as a categorical variable. Therefore, the results may not be interpreted in the context of the World Health Organisation [WHO] definition of higher and lower parity^50^. This limitation notwithstanding, further research may be required to appreciate the possible reasons accounting for the positive association observed in the lower quantiles in Mozambique.

It is also necessary to recognise that mixed findings of the effects of parity on maternal health have been observed in other settings, especially with regards to the relationship between maternal parity and anaemia in pregnancy (AIP). Indeed, while some studies suggest that an increase in parity is associated with higher risks of AIP^51,52^, others reported no significant association between parity and anaemia outcomes^53,54^. Interestingly, related studies revealed that higher parity has positive effects on maternal anaemia outcomes^55,56^. Although these studies focused on AIP, the mixed findings suggest that the impact of parity on maternal anaemia outcomes is complex. Therefore, the results obtained in Mozambique may not necessarily be a deviation from the normal.

An essential strength of this study is that the outcome variable was objectively measured, thereby reducing the possible biases associated with subjective measurements. The use of QR helped to examine the effects of the socio-demographic factors at different points of the Hb concentration, and thus present a comprehensive picture of the effects. Another necessary strength is the use of nationally representative data, making it possible for the results to be generalised to all women of reproductive age in the respective countries. We could not establish causality in this study due to the cross-sectional nature of the data. Also, missing data is an essential limitation of secondary data analysis. However, due to the robust measures put place by DHS to ensure the completeness of their datasets, missing data was not an issue in our study.

## Conclusions

We used quantile regression to examine the effects of socio-demographic factors on maternal Hb concentration. Our analysis demonstrated substantially that the various putative socio-demographic factors have differential effects on maternal Hb concentration at different points of the Hb distribution in all countries. Interventions and programmes to address maternal anaemia must take into account the differential effects of the various socio-demographic factors on Hb concentration throughout the different percentiles of the Hb distribution. It may help identify suitable interventions for groups most in need.

## Data Availability

This study was a re-analysis of existing data that are publicly available from The DHS Program at http://dhsprogram.com/publications/publication-fr221-dhs-final-reports.cfm. Data are accessible free of charge upon registration with the Demographic and Health Survey program (The DHS Program). The registration is done on the DHS website indicated above.

## References

[1] World Health Organization. Anaemia: https://www.who.int/health-topics/anaemia#tab=tab_1 (2018)

[2] World Health Organization. WHO. Nutritional anaemias: tools for effective prevention and control. Nutrition: http://www.who.int/nutrition/publications/micronutrients/anaemias-tools-prevention-control/en/. (2019)

[3] Chaparro CM, Suchdev PS (2019). Anemia epidemiology, pathophysiology, and etiology in low- and middle-income countries. Ann. N. Y. Acad. Sci..

[4] Kassebaum NJ (2016). The global burden of Anemia. Hematol. Oncol. Clin. North Am..

[5] World Health Organization. Global Targets 2025: to improve maternal, infant and young child nutrition: https://www.who.int/nutrition/global-target-2025/en/. (2010).

[6] World Health Organization. Nutrition: Global Nutrition Targets 2025: Anaemia policy brief: https://www.who.int/nutrition/publications/globaltargets2025_policybrief_anaemia/en/. (2014).

[7] Lopez A, Cacoub P, Macdougall IC, Peyrin-Biroulet L (2016). Iron deficiency anaemia. The Lancet..

[8] Young, M.F. Maternal anaemia and risk of mortality: a call for action. *Lancet Glob. Health.* **6,** (2018). 10.1016/S2214-109X(18)30185-2 29571593

[9] Stevens GA (2013). Global, regional, and national trends in haemoglobin concentration and prevalence of total and severe anaemia in children and pregnant and non-pregnant women for 1995–2011: a systematic analysis of population-representative data. Lancet Glob. Health..

[10] United Nations. Sustainable Development Goals: http://www.un.org/sustainabledevelopment/sustainable-development-goals/. (2015)

[11] Ayoya MA (2012). Maternal anaemia in West and Central Africa: time for urgent action. Public Health Nutrition..

[12] van den Broek N (2001). Anaemia in pregnancy in sub-Saharan countries. Eur. J. Obstet. Gynecol. Reprod. Biol..

[13] Tako, E.A. et al. Risk factor for placental malaria and its effect on pregnancy outcome in Yaounde, Cameroon. *Am. J. Trop. Med. Hyg.* **72**, (2005). 15772313

[14] Rahman MM (2016). Maternal anemia and risk of adverse birth and health outcomes in low- and middle-income countries: systematic review and meta-analysis. Am. J. Clin. Nutr..

[15] Black RE (2013). Maternal and child undernutrition and overweight in low-income and middle-income countries. Lancet.

[16] Kavle JA (2008). Association between anaemia during pregnancy and blood loss at and after delivery among women with vaginal births in Pemba Island, Zanzibar Tanzania. J. Health Popul. Nutr..

[17] Christian P (2015). Nutrition and maternal, neonatal, and child health. Semin. Perinatol..

[18] Chowdhury HA (2015). Factors associated with maternal anaemia among pregnant women in Dhaka city. BMC Women’s Health..

[19] Kofie P (2019). Prevalence and associated risk factors of anaemia among women attending antenatal and post-natal clinics at a public health facility in Ghana. BMC Nutrition..

[20] Mockenhaupt FP (2000). Anaemia in pregnant Ghanaian women: importance of malaria, iron deficiency, and haemoglobinopathies. Trans. R. Soc. Trop. Med. Hyg..

[21] Gaillard R (2014). Risk factors and consequences of maternal anaemia and elevated haemoglobin levels during pregnancy: a population-based prospective cohort study. Paediatr. Perinat. Epidemiol..

[22] Hakizimana, D. et al. Identifying risk factors of anemia among women of reproductive age in Rwanda - a cross-sectional study using secondary data from the Rwanda demographic and health survey 2014/2015*. BMC Public Health.* **19,** (2019). 10.1186/s12889-019-8019-z PMC6907339 31829161

[23] Bentley ME, Griffiths PL (2003). The burden of anemia among women in India. Eur. J. Clin. Nutr..

[24] Gobezie, M. et al. Prevalence and Predictors of Maternal Anemia during Pregnancy in Gondar, Northwest Ethiopia: An Institutional Based Cross-Sectional Study. *Anemia*. **108593**, (2014). 10.1155/2014/108593 PMC3942101 24669317

[25] Harding, K.L. et al. Determinants of anaemia among women and children in Nepal and Pakistan: An analysis of recent national survey data. *Matern. Child. Nutr.* **14,** (2018). 10.1111/mcn.12478 PMC6586025 28857410

[26] The DHS Program. Demographic and Health Surveys: http://dhsprogram.com/data/available-datasets.cfm. (2019).

[27] Amugsi, D. A., Dimbuene, Z. T., & Kyobutungi, C. Correlates of the double burden of malnutrition among women: an analysis of cross-sectional survey data from sub-Saharan Africa. *BMJ Open*. ***9*** (2019). 10.1136/bmjopen-2019-029545 PMC6615784 31272983

[28] Ghana Statistical Service (GSS), Ghana Health Service (GHS), ICF Macro. GhanaDemographic and Health Survey (2008). Accra.

[29] Ministry of Health and Social Services (MoHSS), Macro International Inc. Namibia Demographic and Health Survey 2006–07 Windhoek, Namibia and Calverton, Maryland, USA: MoHSS and Macro International Inc. (2008).

[30] Ghana Statistical Service (GSS), Ghana Health Service (GHS), ICF International. Ghana Demographic and Health Survey (2014). Rockville.

[31] National Population Commission (NPC) [Nigeria], ICF International. Nigeria Demographic and Health Survey (2013). Abuja, Nigeria, and Rockville.

[32] National Bureau of Statistics-Kenya, ICF International (2014). KDHS Key Findings.

[33] World Medical Association (2001). Declaration of Helsinki: ethical principles for medical research involving human subjects. Bull. World. Health. Organ..

[34] Koenker R, Bassett G (1978). Regression quantiles. Econometrica..

[35] Amugsi, D.A. et al. Dietary diversity, socioeconomic status and maternal body mass index (BMI): quantile regression analysis of nationally representative data from Ghana, Namibia and Sao Tome and Principe. *BMJ Open*. **6,** (2016). 10.1136/bmjopen-2016-012615 PMC5051549 27678544

[36] DHS. Anaemia among women and children: https://www.dhsprogram.com/pubs/pdf/OD28/12Chapter12.pdf (1999).

[37] Balarajan YS, Fawzi WW, Subramanian SV (2013). Changing patterns of social inequalities in anaemia among women in India: cross-sectional study using nationally representative data. BMJ Open..

[38] Lakew Y, Biadgilign S, Haile D (2015). Anaemia prevalence and associated factors among lactating mothers in Ethiopia: evidence from the 2005 and 2011 demographic and health surveys. BMJ Open..

[39] Friel, J., Qasem, W. & Cai, C. Iron and the Breastfed Infant. *Antioxidants (Basel)*. **7,** 1 (2018). 10.3390/antiox7040054 PMC5946120 29642400

[40] Petraro P (2013). Determinants of anemia in postpartum HIV-negative women in Dar es Salaam Tanzania. Eur. J. Clin. Nutr..

[41] Abioye AI (2016). Iron supplementation affects hematologic biomarker concentrations and pregnancy outcomes among iron-deficient Tanzanian women. J. Nutr..

[42] Jorgensen, J.M. et al. Effect of iron supplementation during lactation on maternal iron status and oxidative stress: A randomised controlled trial. *Matern. Child. Nutr.* **13**, (2017). 10.1111/mcn.12394 PMC6866113 27896921

[43] Abu-Ouf NM, Jan MM (2015). The impact of maternal iron deficiency and iron deficiency anemia on child’s health. Saudi Med. J..

[44] Ejigu BA, Wencheko E, Berhane K (2018). Spatial pattern and determinants of anaemia in Ethiopia. PLoS ONE.

[45] Gebremedhin S, Enquselassie F, Umeta M (2014). Prevalence and correlates of maternal anemia in rural Sidama Southern Ethiopia. Afr. J Reprod. Health..

[46] Derso T, Abera Z, Tariku A (2017). Magnitude and associated factors of anemia among pregnant women in Dera District: a cross-sectional study in northwest Ethiopia. BMC Res. Notes..

[47] Liyew, A.M & Teshale, A.B. Individual and community level factors associated with anemia among lactating mothers in Ethiopia using data from Ethiopian demographic and health survey, 2016; a multilevel analysis. *BMC Public Health.* **775,** 1 (2020) 10.1186/s12889-020-08934-9 PMC7247135 32448212

[48] Selvaraj R (2019). High prevalence of anemia among postnatal mothers in Urban Puducherry: a community-based study. J. Family Med. Prim. Care..

[49] Aliyu MH, Jolly PE, Ehiri JE, Salihu HM (2005). High parity and adverse birthoutcomes: exploring the maze. Women Birth..

[50] Rubio-Álvarez A, Molina-Alarcón M, Hernández-Martínez A (2018). Incidence of postpartum anaemia and risk factors associated with vaginal birth. Women Birth..

[51] Rizk DE, Khalfan M, Ezimokhai M (2001). Obstetric outcome in grand multipara in the United Arab Emirates: a case control study. Arch. Gynecol. Obstet..

[52] Kumari AS, Badrinath P (2002). Extreme grand multiparity: is it an obstetric risk factor?. Eur. J. Obstet. Gynecol. Reprod. Biol..

[53] Fayed HM, Abib SF, Stevens B (1993). Risk factors in extreme grand multiparity. Int. J. Gynecol. Obstet..

[54] Toohey J (1995). The dangerous multipara: fact or fiction?. Am. J. Obstet. Gynecol..

[55] King PA, Duthie SJ, Ma HK (1991). Grand multiparity: a reappraisal of the risks. Int. J. Gynaecol. Obstet..

[56] Silva JP (1992). Grand grand multiparity. J. Obstet. Gynaecol..

